# Exposure and Inequality of PM_2.5_ Pollution to Chinese Population: A Case Study of 31 Provincial Capital Cities from 2000 to 2016

**DOI:** 10.3390/ijerph191912137

**Published:** 2022-09-25

**Authors:** Peiyue Tu, Ya Tian, Yujia Hong, Lu Yang, Jiayi Huang, Haoran Zhang, Xin Mei, Yanhua Zhuang, Xin Zou, Chao He

**Affiliations:** 1 Faculty of Resources and Environmental Science, Hubei University, Wuhan 430062, China; 2 School of Resource and Environmental Sciences, Wuhan University, Wuhan 430079, China; 3 Wuhan Britain-China School, Wuhan 430034, China; 4 Woodsworth College, University of Toronto, Toronto, ON M5S1A9, Canada; 5 Department of Geography, University of Washington, Seattle, WA 98195, USA; 6 Innovation Academy for Precision Measurement Science and Technology, Chinese Academy of Sciences, Wuhan 430077, China; 7 College of Resources and Environment, Yangtze University, Wuhan 430100, China

**Keywords:** PM_2.5_ concentrations, health risk, exposure inequality, GTWR, China

## Abstract

Fine particulate matter (PM_2.5_) exposure has been linked to numerous adverse health effects, with some disadvantaged subgroups bearing a disproportionate exposure burden. Few studies have been conducted to estimate the exposure and inequality of different subgroups due to a lack of adequate characterization of disparities in exposure to air pollutants in urban areas, and a mechanistic understanding of the causes of these exposure inequalities. Based on a long-term series of PM_2.5_ concentrations, this study analyzed the spatial and temporal characteristics of PM_2.5_ in 31 provincial capital cities of China from 2000 to 2016 using the coefficient of variation and trend analyses. A health risk assessment of human exposure to PM_2.5_ from 2000 to 2016 was then undertaken. A cumulative population-weighted average concentration method was applied to investigate exposures and inequality for education level, job category, age, gender and income population subgroups. The relationships between socioeconomic factors and PM_2.5_ exposure concentrations were quantified using the geographically and temporally weighted regression model (GTWR). Results indicate that the PM_2.5_ concentrations in most of the capital cities in the study experienced an increasing trend at a rate of 0.98 μg m^−3^ per year from 2000 to 2016. The proportion of the population exposed to high PM_2.5_ (above 35 μg m^−3^) increased annually, mainly due to the increase of population migrating into north, east, south and central China. The higher educated, older, higher income and urban secondary industry share (SIS) subgroups suffered from the most significant environmental inequality, respectively. The per capita GDP, population size, and the share of the secondary industry played an essential role in unequal exposure to PM_2.5_.

## 1. Introduction

Long-term exposure to high concentrations of urban air pollution not only introduces negative effects on human health, but also threatens sustainable development of the economy and society [1,2,3,4,5,6]. In particular, inequality in exposure to air pollution has recently become an important and timely issue because the health risk of some effects associated with worse conditions of environmental stressors such as air pollution, noise or excessive heat is high for socioeconomically disadvantaged groups [7,8]. This is particularly important in provincial capital cities with strong socioeconomic and air quality gradients, where a local action plan on air pollution can reduce or unintentionally exacerbate inequalities [9,10].

A quantified study on the spatio-temporal variation of the population exposed to different PM_2.5_ concentrations is essential to investigate the inequality of PM_2.5_ exposure in different subgroups and to screen out the main drivers [11]. PM_2.5_ concentration data are a prerequisite for the study to be conducted, and the main methods currently include retrieval of Aerosol Optical Depth as well as actual monitoring [12,13]. In recent years, many comprehensive exposure models have been used to simulate the distribution of populations exposed to different levels of air pollution globally and regionally [14,15]. These models explain the population exposure caused by air pollution to a certain extent but cannot analyze the impact of PM_2.5_ contribution of population change affected by pollution risk. In addition, PM_2.5_ concentrations were significantly related to many socioeconomic factors [7,16,17]. Various spatial econometric method approaches (e.g., spatial lag model, spatial error model, or dynamic spatial panel data model) have been used to explore the relationship between PM_2.5_ concentration and socioeconomic factors [18,19,20]. However, these studies only consider the time dimension and space dimension separately. It is necessary to consider both time and space dimensions. The geographically and temporally weighted regression (GTWR) model incorporates the time effect based on the geographically weighted regression (GWR) model, and thereby the possibility of assessing parametric heterogeneity both in time and space dimensions is provided [21]. Previous studies have shown that the GTWR model has more explanatory power for the spatial and temporal changes in the relationship between air pollution and socioeconomic factors [16,22,23].

Large numbers of studies over the past several decades have been conducted to investigate the inequality of exposure to air pollution among the subgroups of distinct ages, income and ethnicity, which can potentially contribute to environmental health disparities [7,24,25,26,27,28,29,30,31]. For example, based on a 1 km^2^ model, PM_2.5_ and NO_2_ concentrations in Massachusetts over eight years and Census demographic data, Rosofsky firstly quantified inequality between sociodemographic groups exposed to PM_2.5_ and NO_2_ in Massachusetts [29]. They found that certain vulnerable populations, such as blacks, Hispanics and economically deprived people, suffered from inequitable exposures and effects because they were more likely to live in areas with higher concentrations of air pollutants. Bell and Ebisu also investigated the inequality of environmental exposures to PM_2.5_ components that differed by race or ethnicity, age, and socioeconomic status (SES). The results indicated Hispanics, young persons, and lower SES had the highest exposures, respectively. Other researchers also show low-income earners or minorities are exposed to relatively high concentrations of air pollutants [8,32]. Little research on exposure inequality has been carried out in China. Sun et al. found that the poorer people were more likely to suffer from air pollution exposure inequality than the richer people because they invested less in self-protection products [30]. Similarly, the research on disproportionate exposure to PM_2.5_ conducted in Beijing showed that for the highest 1-h concentration, older people (age ≥ 60) and residents with tertiary education were disproportionately exposed to the most PM_2.5_ [28].

However, previous studies have mostly assessed short-term exposure for the entire population, estimated inequality focused on race, ages and income subgroups at a single city scale, ignored the inequality caused by the type of work, gender and education level. In addition, few researchers investigated the drivers of changes in the spatio-temporal distribution of exposure inequality to air pollutants. Therefore, in this study, PM_2.5_ pollution spatio-temporal trends in 31 provincial capital cities in China from 2000 to 2016 were investigated. We used the Exposed Population Contribution Analysis Model (EPCAM) to quantitatively study the populations exposed to different PM_2.5_ concentrations. Then, we combined PM_2.5_ concentration with demographic data to estimate cumulative population weighted average concentrations (CPWAC) in different subgroups of the capital cities and compared the exposure inequality of these subgroups. Additionally, the GTWR model was constructed to quantify the impact of the key socioeconomic factors on PM_2.5_ pollution exposure inequality in time and space dimensions. These findings can provide scientific methods for air pollution exposure and long-term health risk assessments.

## 2. Methods

### 2.1. Study Areas

**Figure 1:**
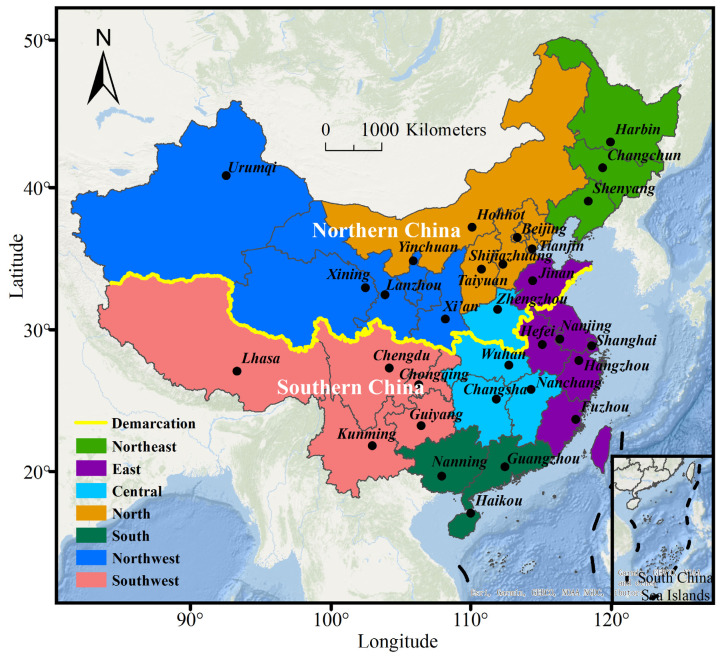
Map of China. (Northern and southern China are divided by the yellow line).

This study analyzed the PM_2.5_ pollution data and socioeconomic data in 31 provincial capital cities of China. These cities are divided into seven regions, including the central, east, north, northeast, northwest, south and southwest China (Figure 1).

### 2.2. PM_2.5_ Concentration

This study analyzed the annual mean concentrations of PM_2.5_ in each provincial capital city from 2000 to 2016. In January 2013, the China National Environmental Monitoring Centre started publishing real-time hourly concentrations of PM_2.5_ monitoring data [33,34]. Therefore, this study processed the 0.01° × 0.01° annual mean global GWR-adjusted PM_2.5_ estimates to obtain the annual average PM_2.5_ concentrations of 31 provincial capital cities in China from 2000 to 2013 using the zonal statistics tool in ArcMap provided by ArcGIS Desktop 10.6. The estimates were calculated by Donkelaar et al., who estimated global PM_2.5_ concentrations using information from satellite-, simulation- and monitor-based sources by applying a Geographically Weighted Regression (GWR) to global geophysically based satellite-derived PM_2.5_ estimates. The resultant PM_2.5_ estimates were highly consistent (R^2^ = 0.81; the slope of 0.82) with out-of-sample cross-validated PM_2.5_ concentrations from monitors [35].

The annual mean levels of PM_2.5_ from 2014 to 2016 were collected from the National Urban Air Quality Real-time Publishing Platform. 

### 2.3. Socioeconomic Data

The socioeconomic data and the population data were collected from the National Bureau of Statistics of the People’s Republic of China (NBSPRC) and the Easy Professional Superior (EPS) data platform. Populations were considered as the sum of people in a given city. Therefore, to assess exposure, we only used the population at the city level corresponding to the mean PM_2.5_ concentrations. The main socioeconomic data include urban area (UA), natural population growth rate, total urban population (UP), population density (PD), urban secondary industry share (SIS), real gross domestic product per capita (GDPPC), education attainment, urban per capita disposable income (UPCDI), rural per capita disposable income (RPCDI), job category, age and gender. We categorized population characteristics into the groups shown in Table 1. 

The sources and download links of the above multi-source datasets are provided in Table 2.

### 2.4. Trend Analysis

Trend analysis is commonly used in temporal dynamic analyses to explore interannual variation characteristics. In this study, a long sequence of *PM*_2.5_ change trends was quantitatively analyzed based on the trend analysis method. Tendencies of *PM*_2.5_ variations can be determined as follows:

(1) $$Trend=\frac{n\times \sum_{i=1}^{n}\left({i\times PM}_{2.5i}\right)-(\sum_{i=1}^{n}i)(\sum_{i=1}^{n}{PM}_{2.5i})\;}{n\times \sum_{i=1}^{n}i^{2}-{\left(\sum_{i=1}^{n}i\right)}^{2}}\;$$

where *PM*_2.5_ is the *PM*_2.5_ concentrations in each city, *n* is the timespan, and *i* is the time unit.

### 2.5. Coefficient of Variation

The spatial heterogeneity of observed concentrations in each city was investigated using a coefficient of variation (*CV*), and it was used to describe the degree of the spatial variations of air pollutant concentrations in Chinese capital cities [36], expressed by:

(2) $$CV=\frac{STD}{\bar{x}}$$

where *STD* and $\bar{x}$ represent standard deviation and mean value.

### 2.6. Contribution Analysis

The increased population exposed to the PM_2.5_ risk in each provincial capital city of China was derived from population migration and natural growth [37], which could be calculated by using the population amount and annual natural population growth rate (*α_i_*), and defined as follows:

(3) $$\Delta {P=P}_{2016}-P_{2000}{=P}_{m}{+P}_{n}\;\;\;\;\;$$

(4) $$P_{n}=\overset{2015}{\underset{i=2000}{\sum }}P_{i}\alpha_{i}$$

(5) $$P_{m}=\overset{2015}{\underset{i=2000}{\sum }}\left[P_{i+1}-P_{i}\left({1+\alpha }_{i}\right)\right]$$

where ΔP represents the change of population that is exposed to *PM*_2.5_ pollution, *Pn* and *Pm* represent the population migration and natural growth, respectively. $\alpha_{i}$ represents the annual natural population growth rate.

### 2.7. Cumulative Population Weighted Average Concentrations (CPWAC)

The cumulative annual mass concentrations of air pollutants in the 31 provincial capital cities were represented by the cumulative annual population weighted average concentrations (*CPWAC*) in all years within each city.

(6) $$CPWAC=\frac{\sum_{i=1}^{n}{(C}_{i}{\times Pop}_{i})}{\sum_{i=1}^{n}{Pop}_{i}}\;$$

where *C* is the mass concentration of the air pollutants. *Pop* is the amount of population. The suffixes *i* represent different years. *n* is the number of all years within each city.

### 2.8. Geographically and Temporally Weighted Regression (GTWR) Model

In this study, the impact of socioeconomic factors on PM_2.5_ concentration was simulated by Geographically and Temporally Weighted Regression model (GTWR). The GTWR captures spatio-temporal heterogeneity based on a weighting matrix referencing both spatial and temporal dimensions [38]. In this study, a GTWR model was fitted using the following structure:

(7) $$\begin{array}{c} {PM}_{2.5i}{=\beta }_{0}\left(\mu_{i}{,v}_{i}{,t}_{i}\right){+\beta }_{1}\left(\mu_{i}{,v}_{i}{,t}_{i}\right){\times UR}_{i}{+\beta }_{2}\left(\mu_{i}{,v}_{i}{,t}_{i}\right)\times {UP}_{i}{+\beta }_{3}\left(\mu_{i}{,v}_{i}{,t}_{i}\right){\times GDPPC}_{i}\\ {+\beta }_{4}\left(\mu_{i}{,v}_{i}{,t}_{i}\right){\times SIS}_{i}{+\beta }_{5}\left(\mu_{i}{,v}_{i}{,t}_{i}\right){\times PD}_{i}+\beta_{6}\left(\mu_{i}{,v}_{i}{,t}_{i}\right){\times UPCDI}_{i}\\ +\beta_{7}\left(\mu_{i}{,v}_{i}{,t}_{i}\right){\times T}_{i}+\varepsilon_{i} \end{array}$$

where *PM*_2.5_*_i_* is the annual surface PM_2.5_ concentration of sample *i*; (*μ_i_*, *υ_i_*, *t_i_*) is the longitude, dimension and time of *i*. *β*_0_ (*μ_i_*, *υ_i_*, *t_i_*) represents the intercepts of *i*. *β*_1_–*β*_6_ are the location-time-specific slopes for UR, UP, GDPPC, SIS, PD, and UPCDI, respectively. The procedure of GTWR model calculation was performed in the GWmodel S (BETA) software (http://gwmodel.whu.edu.cn (accessed on 30 May 2021)), where the type of spatial kernel function selected during the model implementation was Bisquare, the type of bandwidth search was AICc, and the type of distance criterion was CRS type. Meanwhile, the spatio-temporal heterogeneous characteristics of the independent variables were examined with the OLS model in the advanced settings of the GTWR model.

## 3. Results

### 3.1. PM_2.5_ Pollution Characteristics

**Figure 2:**
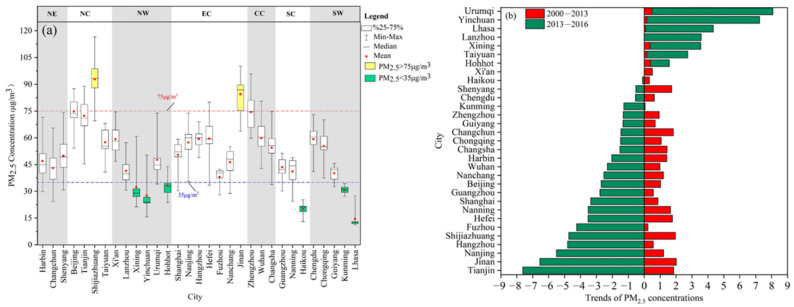
Annual average PM_2.5_ concentration (**a**) and (**b**) the trends of PM_2.5_ concentrations in provincial capital cities from 2000 to 2016.

**Figure A1:**
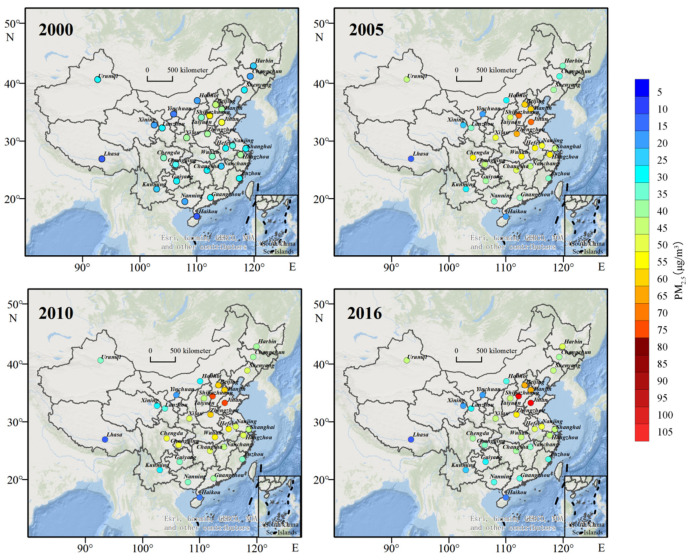
Spatial distribution of PM_2.5_ concentration in 31 provincial capital cities during 2000–2016.

Figure 2a shows the annual average of PM_2.5_ concentrations in 31 provincial capital cities during the study periods. The annual average concentrations of PM_2.5_ ranged from 14.46 ± 5.19 μg m^−3^ (Lhasa) to 92.75±12.29 μg m^−3^ (Shijiazhuang). The PM_2.5_ concentrations in all the capital cities exceeded the CAAQS (Chinese Ambient Air Quality Standards (GB3095-2012)) Grade I standard (15 μg m^−3^), and exceeded the Grade II standard (35 μg m^−3^) except Xining, Yinchuan, Hohhot, Haikou, Kunming, and Lhasa. Annual average PM_2.5_ concentrations in 31 provincial capitals increased to varying degrees from 2000 to 2016, and PM_2.5_ pollution increased significantly (Figure A1, Appendix A). Therefore, the temporal variation of annual PM_2.5_ concentrations in each provincial capital city was quantified in two time periods (Figure 2b). Overall, the annual average of PM_2.5_ over the whole study cities experienced a minor increasing trend at a rate of 0.94 μg m^−3^ per year except Lanzhou and a small CV of 0.16 from 2000 to 2013. However, a majority of the capital cities, 74%, had a large decreased trend at a rate of 2.86 μg m^−3^ per year from 2013 to 2016, which showed that the focus and measures exerted on air pollution control in recent years may have gained good effect [20]. Specifically, the “Air Pollution Prevention and Control Action Plan” set definite targets for decreasing PM_2.5_ concentrations in most eastern regions and other heavily polluted regions. Additionally, the changing trends were different from city to city, with strong spatial variability (Appendix A). Spatially, in comparison to other cities, towns in eastern and northern China had greater magnitudes of decreases in PM_2.5_ concentrations (Figure 2b). Cities with an increase in PM_2.5_ concentrations mainly were located in northwest China, where cities experienced an increasing trend (Figure 2b) from 2013 to 2016.

### 3.2. The Populations Exposed to Different PM_2.5_ Concentrations

**Figure 3:**
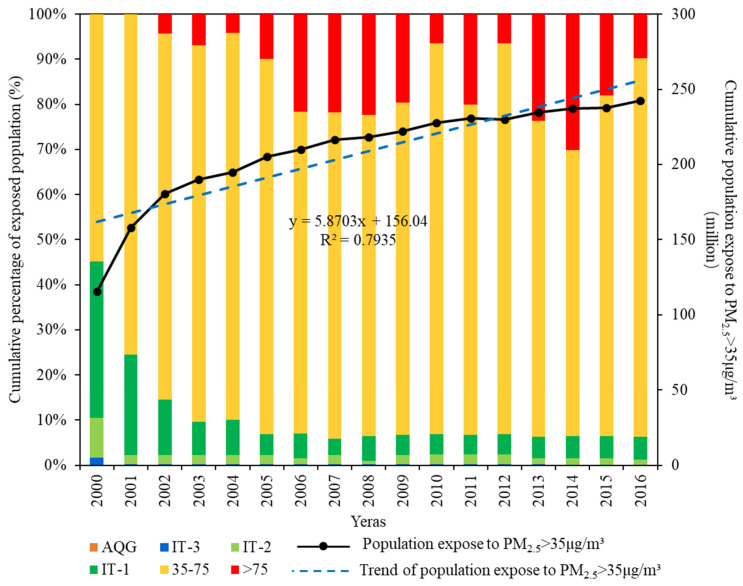
Changes in the proportion of the population exposed to different PM_2.5_ concentrations in 31 provincial capital cities, 2000–2016; AQG, IT-3, IT-2, IT-1 refer to WHO air quality guidelines (<10 μg m^−3^), Interim target 3 (10–15 μg m^−3^), Interim target 2 (15–25 μg m^−3^), Interim target 1 (25–35 μg m^−3^).

Based on population data statistics, we quantified the temporal changes in the proportions of populations exposed to different PM_2.5_ concentrations in provincial capital cities of China from 2000 to 2016 (Figure 3). In general, the proportion of the total population exposed to PM_2.5_ less than 35 μg m^−3^ decreased from 45.14% in 2000 to 6.44% in 2008 and 6.35% in 2016, whereas the proportion exposed to PM_2.5_ over 35 μg m^−3^ increased from 54.86% in 2000 to 93.56% in 2008 and 93.65% in 2016. These results showed that an increasing fraction of the population suffered from increasingly serious PM_2.5_ pollution and that fewer people experienced light PM_2.5_ pollution. Therefore, over the past 17 years, an increasing proportion of the population has mainly concentrated in those areas with concentrations of 35–75 μg m^−3^ of PM_2.5_. In summary, the most significant change was the increasing population susceptible to experiencing high PM_2.5_ concentrations, and a declining proportion of the population experiencing low PM_2.5_ concentrations. In addition, the population exposed to PM_2.5_ over 35 μg m^−3^ will increase following the increase of PM_2.5_ pollution in the future years (Figure 3). 

**Figure A2:**
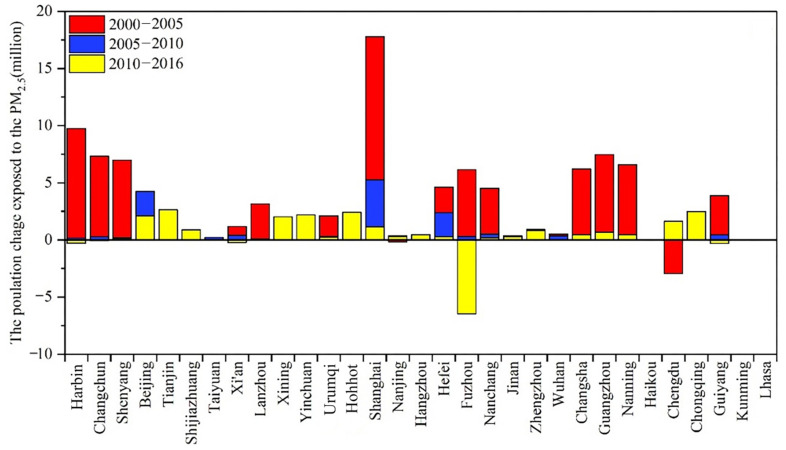
The change of population in three terms of 2000–2005, 2005–2010 and 2010–2016.

From 2000 to 2016, a total of 3561.62 million people were exposed to PM_2.5_ over 35 μg m^−3^ (i.e., potential risk) while only 406.55 million were exposed to PM_2.5_ less than 35 μg m^−3^ in 31 provincial capital cities of China. All populations of Beijing, Tianjin, Taiyuan, Shijiazhuang, Chengdu, Chongqing, Wuhan, Zhengzhou, Xi’an, Hangzhou, Nanjing, and Jinan were exposed to the potential risk. More than 90% of the population in the provincial capital cities of Hefei (95%) and Shanghai (92%) in east China, Urumqi (95%) in northwest China, Guangzhou (95%) in south China, and Changsha (95%) in central China, were exposed to the potential risk. In contrast, the population exposed to the potential risk in Hohhot, Xining and Yinchuan located in northwest China was less than 30%. In addition, all populations of Haikou in south China along with Kunming and Lhasa in southwest China were exposed to PM_2.5_ less than 35 μg m^−3^. The population exposed to the potential risk was increased in all provincial capital cities except Chengdu during the study periods. The exposed population of most provincial capital cities increased in three different periods, and the average population changes in 2000–2005, 2005–2010 and 2010–2016 were 2.90 million, 0.73 million and 0.47 million, respectively (Figure A2, Appendix A). These results showed that the population exposed to PM_2.5_ over 35 μg m^−3^ increased, but its growth rates declined. Spatially, the population exposure was mainly found in the east (57.04 million in 2016) and north (51.84 million in 2016). 

**Figure 4:**
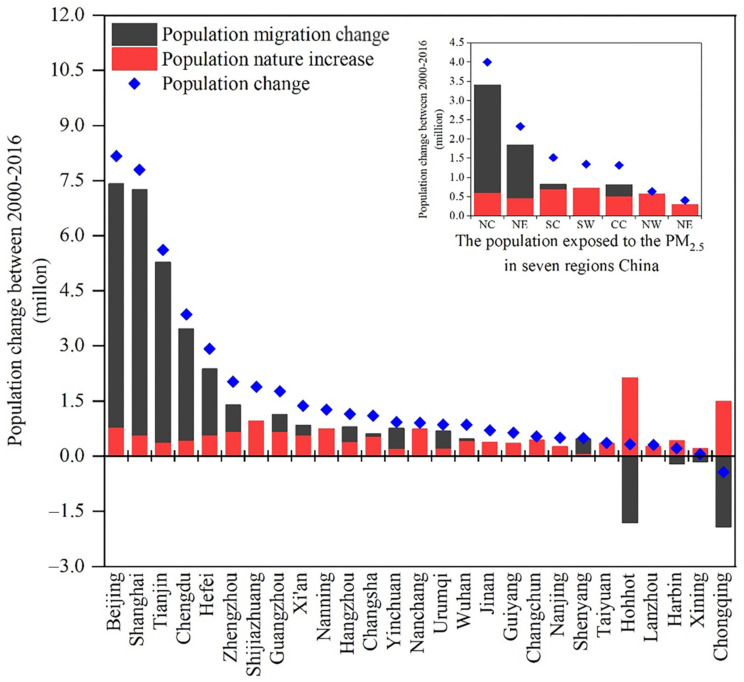
The contributions of migration and natural increase in the population exposed to PM_2.5_ pollution during 2000–2016.

The increased population exposed to the PM_2.5_ risk in each city was derived from population migration and natural growth. As shown in Figure 4, the contributions of exposed population were increased, mainly due to the increase of migration of population in north, east, south and central China. Specifically, population sizes of two Chinese northern cities, Beijing and Tianjin, as well as one eastern city, Shanghai, in 2016 have increased by 8.16, 7.79 and 5.61 million, respectively, compared to 2000, with over 90% of population movement derived from migration. Likewise, from 2000 to 2016, the average increments of population exposed to PM_2.5_ in 10 other cities exceed 1,000,000, including three western cities (Yinchuan, Urumqi and Xi’an), three eastern cities (Hefei, Hangzhou and Nanjing), three central cities (Zhengzhou, Changsha and Wuhan) and one southern city (Guangzhou), and above 60% of this increased population was from migration. These findings indicate that migration could promote the rise of the population exposed to PM_2.5_ and increasing population size would aggravate PM_2.5_ pollution in urban areas. In contrast, the contribution of the migrant population to the exposed population played a negative role in Harbin and Changchun in northeast China, Lanzhou, Xining and Hohhot in northwest China and Chongqing in southwest China (Figure 4). 

### 3.3. Exposure Inequality

**Figure 5:**
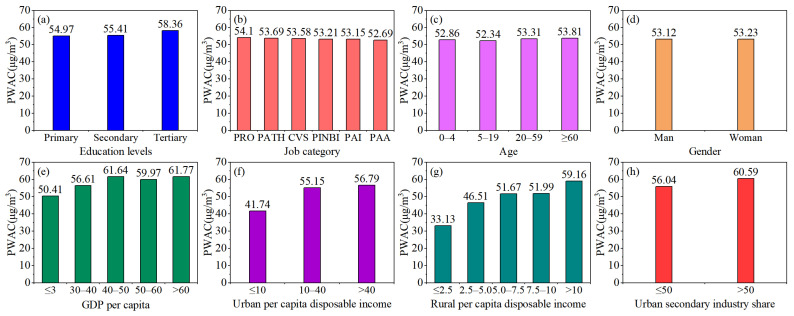
Cumulative annual average population weighted exposures to PM_2.5_ concentrations for different population subgroups: (**a**) Education levels, (**b**) Job category, (**c**) Age, (**d**) Gender, (**e**) GDP per capita, (**f**) Urban per capita disposable income, (**g**) Rural per capita disposable income, (**h**) Urban secondary industry share.

The cumulative annual population weighted exposure concentrations to PM_2.5_ for each subgroup were calculated (Figure 5). For all subgroups, the cumulative annual population weighted exposure concentrations of SIS subgroups (58.31 μg m^−3^) were the highest, while RPCDI subgroups (48.49 μg m^−3^) experienced the lowest exposure to PM_2.5_. Specifically, the annual population weighted exposure concentrations to PM_2.5_ of SIS, GDPPC, education, job category, gender, age, UPCDI, and RPCDI subgroups were 58.31, 58.08, 56.25, 53.40, 53.18, 53.08, 51.23 and 48.49 μg m^−3^, respectively. These were slightly lower than the average for capital cities (0.52–8.27 μg m^−3^), except for SIS and GDPPC subgroups.

For the education subgroups, the estimated average exposure for people with tertiary education was the highest (58.36 μg m^−3^), followed by those with secondary (55.41 μg m^−3^) and primary (54.97 μg m^−3^) education (Figure 5a). This indicated that the exposure levels increased with education level improvement. However, among the job category subgroups, the exposure level for the professionals category (54.10 μg m^−3^) was the highest and less than the average for capital cities (2.67 μg m^−3^ lower). The population weighted exposure concentrations of PATH, CVS, PINBI and PAI in the subgroup of job categories to PM_2.5_ had similar exposure levels, except PAA categories, in which the average annual population weighted exposure concentrations were 53.69, 53.58, 53.21 and 53.15 μg m^−3^, respectively (Figure 5b). For the age subgroups, the adults (20–59, 53.31 μg m^−3^) and older people (age ≥ 60, 53.81 μg m^−3^) suffered from greater exposure to PM_2.5_. Additionally, among age subgroups, the exposure concentrations of adolescents (5–19) were the lowest and slightly lower than the average of capital cities (4.42 μg m^−3^ lower) during the study period. The exposure concentrations of young children (age ≤ 4, 52.86 μg m^−3^) were also slightly lower than the average exposure level (Figure 5c). Differences in gender were indistinguishable, and the exposure concentrations of women were slightly higher than men’s (0.11 μg m^−3^ higher) (Figure 5d).

The highest GDPPC (over 60,000 Yuan) subgroups were found to have the highest average exposure to PM_2.5_ concentration among all subgroups, with 5.01 μg m^−3^ higher than the capital cities annual average for PM_2.5_ exposures, while the lowest GDPPC category groups were exposed to the lowest PM_2.5_ concentrations (Figure 5e). This means that the population weighted exposure generally increased with an increasing of GDPPC. A similar inequality was found in income groups. Residents with an annual income of more than RMB 40,000 in urban areas had the highest average exposures, while those who earned more than RMB 10,000 per year in rural areas had the highest ones (Figure 5f,g). Annual average population weighted exposures to PM_2.5_ concentration for the highest income of UPCDI and RPCDI groups (greater than RMB 40,000 and RMB 10,000) were 0.03 and 2.40 μg m^−3^, which were more than the capital cities average. Among the SIS subgroups, the lower SIS groups (less than 50%) had the lowest exposure levels (56.04 μg m^−3^) and were lower than the city average (0.73 μg m^−3^ lower) during the study period (Figure 5h).

### 3.4. Economic Effectiveness of Exposure Inequality

**Figure 6:**
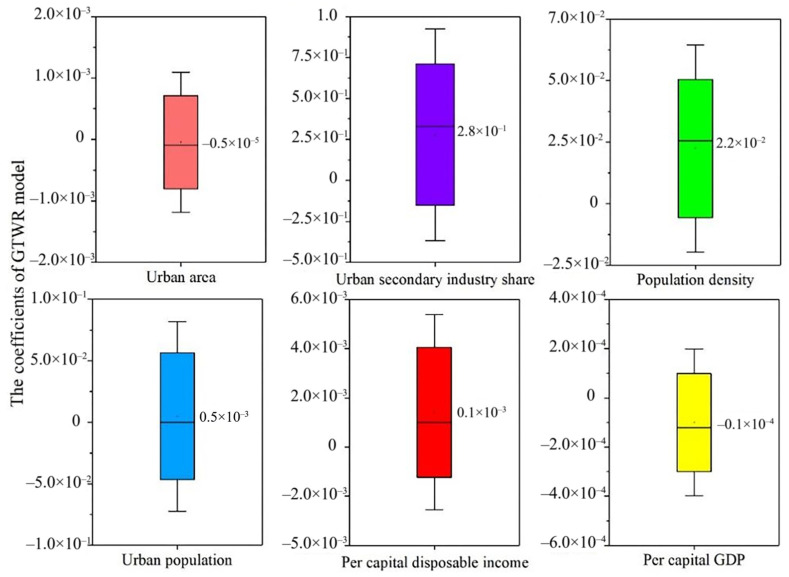
The estimation coefficients descriptive statistics of the GTWR model.

PM_2.5_ exposure was significantly related to many socioeconomic factors [10,17,24]. GTWR model (see Appendix A for model assessment process) was used to study the spatial heterogeneity of the relationship between socio-economic factors and PM_2.5_ concentration. The results from the GTWR model showed that all variables, except for urban area, were significantly related to PM_2.5_ concentrations. Among all the variables, urban secondary industry share (0.279) was the most important predictor, and population density (0.022) was the second most important predictor (Figure 6). It was indicated that the secondary industry share and population contributed to the change of urban PM_2.5_ concentration.

**Figure 7:**
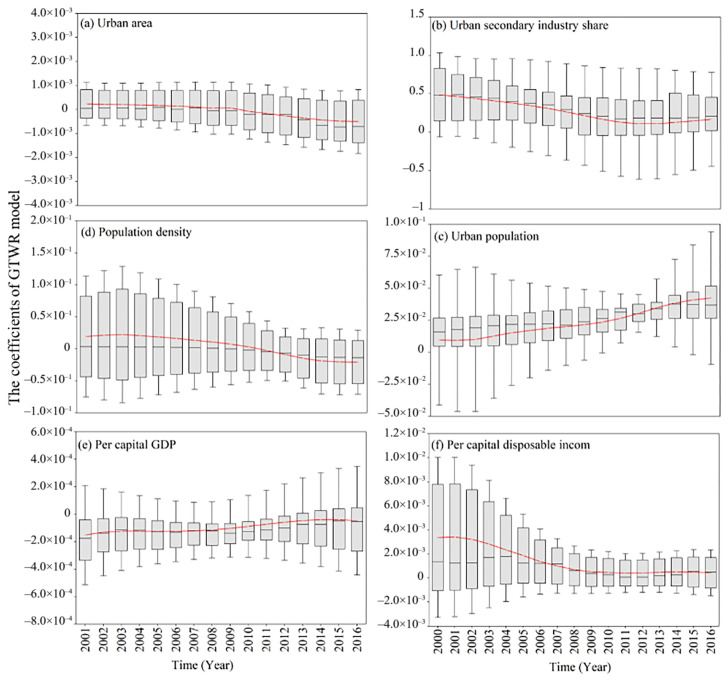
Temporal distribution of estimated coefficients of GTWR model. The red line represents the variation trend of the average coefficient of the GTWR model.

According to Figure 7, the impacts of the urban secondary industry share, population density, urban population and per capita disposable income on the urban PM_2.5_ concentration in most years were positive, while urban area and per capita GDP had negative effects on PM_2.5_ concentration, that is, if the share of secondary industry, population size and population density increase, the change rate of urban PM_2.5_ emissions will be greater, making the urban PM_2.5_ concentration increase. In addition, the trend of the coefficient of the share of the secondary industry (Figure 7b), population density (Figure 7c) and per capita disposable income (Figure 7f) gradually decreased from 2005 to 2016. This indicated that their impacts on PM_2.5_ concentration were getting lower, which was mainly attributed to China’s urban population policy and industrial structure adjustment. On the contrary, sometimes the increased urban population (Figure 7d) and per capita GDP (Figure 7e) have a positive contribution to PM_2.5_ concentration, because, with the further process of industrialization and urbanization, the proportion of secondary industry in GDP has increased, resulting in increased demand for energy consumption [39].

**Figure 8:**
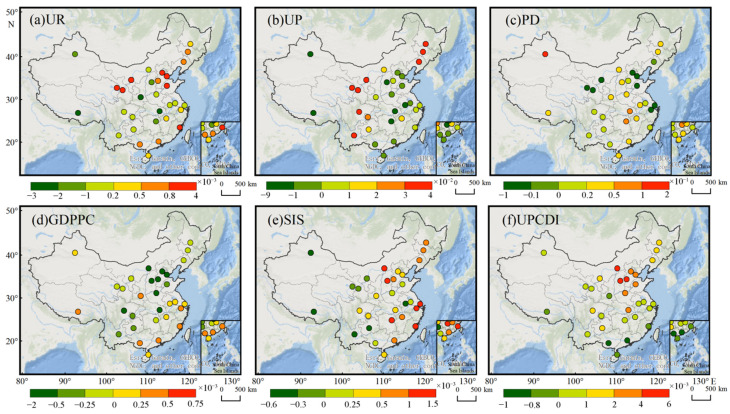
The estimated coefficients of GTWR in 31 provincial capital cities.

Figure 8 shows the spatial distribution of different driver factor coefficients estimated by the GTWR model. There was significant spatial heterogeneity in coefficients of each socioeconomic factor except urban area. The highest coefficients were urban population (0.066), population density (0.062), GDP per capita (0.0003), secondary industry share (0.769) and per capita disposable income (0.005) estimated by GTWR were distributed in northwest, central, south, and north provincial capitals, respectively. This shows that population size and density are important driving factors for PM_2.5_ concentration in cities in the northwest and central China, while secondary industry share and per capita income play an important role in PM_2.5_ concentration in cities in northern China. Specifically, the urban population and population density promoted the increase of PM_2.5_ emission, with estimated coefficients of 0.014 and 0.018, respectively. Compared with other provincial capital cities, cities in the north, east and southwest of China had a higher urban population and population density coefficient, which indicated that the more densely populated the more important influence on PM_2.5_ concentration in a certain area. Higher population density means more energy consumption and pollution emissions, as well as a decrease of green space associated with densification that can reduce the city’s capacity to mitigate air pollution [20,40,41].

Among all the driving factors, per capita GDP played an important role, and over 60% of provincial capital cities per capita GDP had a negative impact on urban PM_2.5_ concentration. Especially, for economically developed regions, such as the Beijing-Tianjin-Hebei region (BTH) and the Pearl River Delta region (PRD), the per capita GDP had a negative impact on the urban PM_2.5_ concentration. On the contrary, for the cities with low economic levels, such as Xining, Yinchuan, Lanzhou, Harbin, Changchun, Shenyang and Lhasa, the per capita GDP had a significant positive impact on the urban PM_2.5_ concentration. The results showed that the spatial difference in PM_2.5_ concentration would cause regional economic inequality. In contrast, per capita disposable income had a positive impact on urban PM_2.5_ concentration in many cities, except for Xi’an (−0.0001), Lhasa (−0.0001), Fuzhou (−0.0003), Haikou (−0.0004), Nanning (−0.0008) and Guangzhou (−0.001). Another important driving factor that can affect urban PM_2.5_ concentration is urban second industry share. Our results showed that, except for Lanzhou (−0.14), Xining (−0.18), Yinchuan (−0.12), Hefei (−0.60), Kunming (−0.50), Guiyang (−0.29), Urumqi (−0.57) and Lhasa (−0.33), the share of the secondary industry had a significant positive impact on PM_2.5_ concentration. Spatially, compared with other provincial capital cities, the share of the secondary industry had a more significant impact on PM_2.5_ concentration in cities in the north, central and eastern China (Table A2, Appendix A). In the process of industrialization and urbanization, the secondary industry accounted for about 40% of GDP, which led to a surge in energy consumption, and at present, the main fuel consumption in most cities is fossil fuels, thus, a large amount of fossil energy consumption produces a large amount of PM_2.5_ emissions and dust emissions [26,39,42].

## 4. Discussion

### 4.1. Differences in Spatio-Temporal Distribution of PM_2.5_

This study analyzed the annual average PM_2.5_ spatio-temporal distribution during 2000–2016 in 31 provincial capital cities of China. The higher PM_2.5_ concentrations primarily occurred in northern, eastern, and central China. The highest annual mean PM_2.5_ concentrations occurred in Shijiazhuang, and the annual mean PM_2.5_ concentrations exceed 75 μg m^−3^ during 2000–2016, except in 2000 and 2001, which was mainly produced by fossil fuel combustion, industrial emissions, and floating dust from building sites. Coal-burning is the most important pollution source, accounting for 28.5% of local emitted PM_2.5_, while PM_2.5_ mass concentrations from industrial emissions and floating dust accounted for 25.2% and 22.5% of local emitted PM_2.5_ in Shijiazhuang, respectively [43]. Additionally, unfavorable meteorological conditions such as weak winds and vertical diffusion and low mixing layer height are the main factors leading to long-term severe air pollution at the low atmosphere in the North China Plain, especially Shijiazhuang and Jinan [44]. The lowest annual mean PM_2.5_ concentrations occurred in Lhasa (14.46 + 5.19 μg m^−3^) which is lower than 35 μg m^−3^ from 2000 to 2016, mainly due to fewer coal-based industries or more favorable meteorological conditions for pollution dispersion than other areas, resulting in these places having lower levels of air pollution [45]. The change rates of PM_2.5_ concentration in three terms of 2000–2005, 2005–2010 and 2010–2016 were 31.41%, −0.08% and −0.16% respectively, which showed that the focus and measures exerted on air pollution control in recent years may have been very effective [20]. Specifically, the “Air Pollution Prevention and Control Action Plan” set definite targets for decreases in PM_2.5_ concentrations for most eastern regions and other heavily polluted regions.

### 4.2. Contribution of Population Mobility to Urban PM_2.5_ Pollution

Previous studies have shown that with the rapid urbanization and industrialization in China, the population exposed to the potential risks of PM_2.5_ was sharply increasing [46,47]. Our results show that over the past 17 years, an increasing proportion of the population was mainly concentrated in areas with PM_2.5_ concentrations of 35–75 μg m^−3^. In addition, more and more people in rural area have migrated to cities, especially where infrastructures are well-developed and economically developed cities are located, such as Beijing, Shanghai, Tianjin and Guangzhou. Chinese urbanization strategy has aimed to increase the urban population from 52% in 2012 to 60% in 2020, which means that at least 100 million people have migrated to cities [48]. This will lead to further urban expansion and larger urban populations imposing additional pressure on urban air quality and PM_2.5_ concentrations than before. Meanwhile, a huge amount of population migration from rural to urban means more energy consumption and pollution emissions, as well as a decrease in the area of green space associated with densification that can weaken the city’s capacity to mitigate air pollution [20,40,41]. Therefore, more reasonable local regulations are needed to be conducted, such as strict limitation of industrial air pollutant emissions, upgrading the gasoline quality ahead or together with improvements in vehicle emission standards [10,45]. The census data used in the assessment of PM_2.5_ exposure are based on the assumption that the population distribution is stationary. In reality, people will be in different places and exposed to different PM_2.5_ environments across different times [49]. Although uncertainty in static-based assessments has been discussed in previous studies, this uncertainty is smaller in the assessment of PM_2.5_ exposure for long time series.

### 4.3. Differences in Exposure and Inequality

Exposure inequality to air pollution means that some impacts related to air pollution are relatively high for socially disadvantaged groups. For the education levels subgroup, the exposure concentrations for people with a tertiary education were the highest, while people with a primary education experienced the lowest levels. This can be explained by the fact that over 80% of the lower education population inhabited the southwest areas with relatively low PM_2.5_ concentration. On the other hand, the higher education groups were often engaged in high-level occupations and had a high income, so they can afford excessive prices and live in the central urban areas where good infrastructure, convenient transportation, good educational and medical institutions are provided, as they are exposed to higher PM_2.5_ concentration. By contrast, residents with low education and low income usually live around the central areas because they cannot afford high housing prices [28,50]. For job category subgroups, public government employees and tertiary industry service personnel, due to the friendly office environment and advanced air purification equipment, had a lower risk of exposure to PM_2.5_ concentrations than workers and professional technicians who had been engaged in the manufacturing industry for a long time.

Among the age subgroups, children and older people were the most unfairly exposed population, which was consistent with the result found by Ouyang et al. [28]. For GDPPC and SIS subgroups, people with higher per capita GDP and located in industry-led industries were more likely to be exposed to high PM_2.5_ risks. These are mainly because economic development in most Chinese provincial capital cities tend to rely on coal-based secondary industries, and coal is the dominating energy source for the secondary industry in China. As a result, the fast growth of the secondary industry caused a huge increase of coal consumption. As Hao and Liu pointed out, the huge coal consumption caused by the development of secondary industry in recent years was an important reason for the rise of urban PM_2.5_ concentrations [19]. For income subgroups, including urban and rural residents, the cumulative population weighted exposure generally increased with an increasing per capita disposable income. For urban residents, higher income people (UPCDI >25) were mainly concentrated in the north and east areas or lived in urban core areas. The distribution characterization of higher income people was in line with that of annual PM_2.5_ concentration, which resulted in the most disproportionate exposure for higher income residents. In contrast, for rural residents, the high-income group was mainly from the population who migrated from rural to urban areas. Most of them were mainly engaged in construction and industrial industries, and construction dust and industrial emissions were the main sources of particulate pollution [51,52].

### 4.4. Implications and Limitations

This research examined the characteristics of PM_2.5_ pollution exposure and explored the inequality of PM_2.5_ exposure in different population subgroups, and discussed the driving factors of unequal exposure in 31 Chinese capital cities from 2000 to 2016. This study demonstrated the importance of multiple temporal scales. It provided evidence for residential exposures to PM_2.5_, which is helpful for the government to identify the exposed subgroups and to take protective measures for these residents. However, there are also some limitations in our research. Firstly, the data of PM_2.5_ concentration used in this study were from 2000 to 2016, while population census data were investigated in 2000 and 2010. To study the exposure inequality of PM_2.5_ to different population subgroups, we only used 2000 and 2010 census data. Although the period of census data does not match that of PM_2.5_ concentration, the results of exposure inequality for PM_2.5_ are small. Secondly, the trends of PM_2.5_ concentration and population were changing with time, so the exposure level and exposure inequality calculated in this study may not represent future exposures. Thirdly, when investigating the impacts of driving factors on PM_2.5_ exposure, this study did not consider the impacts of natural variances.

## 5. Conclusions

In this study, we found that the average annual PM_2.5_ over the whole study of capital cities experienced a minor increase at a rate of 0.94 μg m^−3^ per year, except Lanzhou and a small CV with 0.16 from 2000 to 2013. However, a majority of the capital cities, 74%, saw a sharp decrease at a rate of 2.86 μg m^−3^ per year from 2013 to 2016. According to the variation in temporal scales, an increasing number of people suffered from increasingly serious PM_2.5_ pollution and fewer people experienced less PM_2.5_ pollution. The contributions of increased exposure population are mainly caused by the increase of migrating population in north, east, south and central China. Additionally, the exposure and inequality among different subgroups showed distinct differences. Overall, older people, higher income and higher SIS subgroups suffered from the greater environmental inequality, respectively. At the same time, gender and job category groups had similar exposure levels. In addition, the cumulative population-weighted exposure and inequality level increased with the increasing education level. Finally, we established nonlinear and the GTWR model to analyze the economic effectiveness of exposure inequality. The results showed that per capita GDP, population size and the share of the secondary industry played an important role in exposure inequality to PM_2.5_.

## Figures and Tables

**Table 1:** The population characteristics of each population subgroup.

Population Subgroup	Groups
Education	Primary
	Secondary
	Tertiary
Per capita GDP (thousands of Yuan)	≤30
	30–40
	40–50
	50–60
	>60
The urban secondary industry share (%)	≤50
	>50
Urban per capita disposable income (thousands of Yuan)	≤10
	10–40
	>40
Rural per capita disposable income (thousands of Yuan)	≤2.5
	2.5–5.0
	5.0–7.5
	7.5–10
	>10
Job category	Professionals (PRO)
	Practitioners in third industry (PATH)
	Civil servants (CVS)
	Principal of national bureaus and institutions (PINBI)
	Practitioners in industry (PAI)
	Practitioners in agriculture (PAA)
Age (years)	0–4
	5–19
	20–59
	≥60
Gender	Man
	Woman

**Table 2:** The list of datasets with their sources and download links.

Category	Source	Accessed Date	Uniform Resource Location
2000–2013 PM_2.5_	Atmospheric Composition Analysis Group Website of Dalhousie University	11 May 2021	http://fizz.phys.dal.ca/~atmos/martin/?page_id=140
2014–2016 PM_2.5_	National Urban Air Quality Real-time Publishing Platform	11 May 2021	http://106.37.208.233:20035/
Socioeconomic	EPS	30 May 2021	http://olap.epsnet.com.cn/index.html
Population	NBSPRC	30 May 2021	http://data.stats.gov.cn

**Figure A3:**
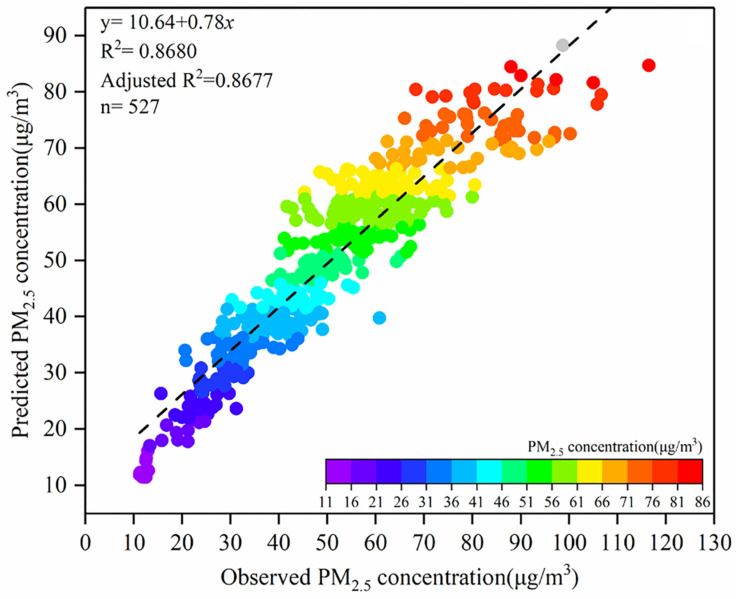
The predicted and observed PM_2.5_ concentration.

## Data Availability

Data used in this paper can be obtained from Chao He (hechao@yangtzeu.edu.cn) upon request.

## Appendix A

### Appendix A.1. Spatial Patterns and Variations in PM2.5

This study analyzed the annual average PM_2.5_ mass concentrations during 2000–2016 in 31 provincial capital cities. Figure A1 shows the annual averages and spatial distribution of PM_2.5_ concentrations in 31 provinces capital cities during 2000–2016. The higher concentrations of PM_2.5_ primarily occurred in northern, eastern, and central China, which mainly resulted from anthropogenic emissions including coal and biomass burning, industrial and motor vehicles emissions, etc. All of the provincial capital cities exceeded WHO air quality guidelines (AQG), and only 3.23%, 3.23%, and 12.90% of the cities met WHO interim target-3 (IT-3, 10–15 μg/m^3^), interim target-2 (IT-2, 15–25 μg/m^3^) and IT-1 thresholds. These results indicate that only 19.25% of the urban population was exposed to annual mean PM_2.5_ concentrations less than the WHO IT-1 limit of 35 μg/m^3^, and 80.65% of the urban population was exposed to annual mean PM_2.5_ concentrations exceeding the WHO IT-1 threshold.

### Appendix A.2. The Change of Population in Three Terms of 2000–2005, 2005–2010, and 2010–2016

As shown in Figure A2, the exposed population of mostly provincial capital cities increased in three different time periods, and the average population changes in 2000–2005, 2005–2010, 2010–2016 were 2.90 million, 0.73 million and 0.47 million, respectively. Our research shows that the population exposed to PM_2.5_ over 35 μg/m^3^ is still increasing; however, its growth rate is becoming slower and slower. Spatially, the population exposure was mainly found in East (57.04 million in 2016) and North (51.84 million in 2016) China, and the increased population exposure from 2000 to 2016 was primarily due to the increase in the exposed population of East China.

### Appendix A.3. GTWR Model Evaluation Process

To better understand the influence of socio-economic factors on urban PM_2.5_ concentration, the GTWR model was used to further study the spatial heterogeneity of the relationship between socio-economic factors and PM_2.5_ concentration. To examine the existence of multicollinearity between the explanatory variables before conducting the regression analysis, this study performed multicollinearity tests for each explanatory variable with SPSS 25.0 software, which showed that the variance inflation factors (VIF) for UR, UP, GDPPC, SIS, UPCDI and PD were 2.44, 1.73, 1.17, 1.06, 2.57 and 2.52, respectively. All of these are less than 10, indicating that there was no significant effect of multicollinearity between the explanatory variables, which allows for the regression analysis in the next step. The GTWR model global regression results show that the GTWR has a bandwidth of 0.1150, with a residual sum of squares of 33,007.9, an AICc of 3822.93, a spatio-temporal distance ratio of 0.3594, and a goodness-of-fit (R^2^Adjusted) of 0.8316. These results indicated that the GTWR model uses fewer parameters to obtain regression results closer to the valid values (Table A1 and Table A4).

**Table A1:** Multicollinearity Test.

Independent Variables	t	VIF
UR	−4.78	2.44
UP	−1.38	1.73
GDPPC	5.28	1.17
SIS	5.49	1.06
PD	9.75	2.52
UPCDI	6.41	2.57
Dependent Variable: PM_2.5_		

**Table A2:** The estimated coefficients of GTWR in seven regions.

Region	UR	UP	PD	GDPPC	SIS	UPCDI
Northeast	5.69 × 10^−4^	6.63 × 10^−2^	1.74 × 10^−2^	−1.77 × 10^−4^	6.68 × 10^−1^	1.18 × 10^−3^
North	9.78 × 10^−4^	−2.47 × 10^−3^	3.08 × 10^−3^	−1.26 × 10^−3^	7.69 × 10^−1^	4.64 × 10^−3^
Northwest	9.63 × 10^−4^	3.79 × 10^−2^	−5.20 × 10^−3^	−2.15 × 10^−4^	1.39 × 10^−1^	1.39 × 10^−3^
East	4.22 × 10^−4^	6.05 × 10^−5^	−8.87 × 10^−4^	1.11 × 10^−4^	5.32 × 10^−1^	5.63 × 10^−4^
Central	−1.14 × 10^−3^	−1.21 × 10^−2^	6.23 × 10^−2^	−5.82 × 10^−4^	4.74 × 10^−1^	2.41 × 10^−3^
South	5.61 × 10^−4^	−3.74 × 10^−3^	3.61 × 10^−2^	3.37 × 10^−4^	3.36 × 10^−1^	−7.68 × 10^−4^
Southwest	−3.88 × 10^−4^	1.01 × 10^−2^	1.40 × 10^−2^	−2.92 × 10^−4^	−1.02 × 10^−1^	8.01 × 10^−4^

**Table A3:** List of abbreviations.

Abbreviation	Description
BTH	Beijing-Tianjin-Hebei
CAAQS	Chinese Ambient Air Quality Standards
CPWAC	Cumulative population weighted average concentrations
CV	Coefficient of variation
CVS	Civil servants
EPCAM	Exposed Population Contribution Analysis Model
EPS	Easy Professional Superior
GDP	Gross Domestic Product
GDPPC	Gross domestic product per capita
GTWR	Geographically and temporally weighted regression
GWR	Geographically weighted regression
NBSPRC	National Bureau of Statistics of the People’s Republic of China
PAA	Practitioners in agriculture
PAI	Practitioners in industry
PATH	Practitioners in third industry
PD	Population density
PINBI	Principal of national bureaus and institutions
PM_2.5_	Fine particulate matter
PRD	Pearl River Delta
PRO	Professionals
RPCDI	Rural per capita disposable income
SES	Socioeconomic status
SIS	Urban secondary industry share
UA	Urban area
UP	Urban population
UPCDI	Urban per capita disposable income

**Table A4:** GTWR Diagnostic Information.

Index	Value
Bandwidth	0.114996
Residual Squares	33,007.9
Sigma	7.91414
AICc	3822.93
R^2^	0.833237
R^2^Adjusted	0.831636
Spatio-temporal Distance Ratio	0.359363

**Table A5:** Analysis of variance (ANOVA).

	Sum of Squares	Degrees of Freedom	Mean Square	F-Statistic	Significance
Between Groups	314.560	7	44.937	1.587	0.191
Within Groups	622.797	22	28.309		
Total	937.358	29			
